# Designing Dual-functionalized Gels for Self-reconfiguration and Autonomous Motion

**DOI:** 10.1038/srep09569

**Published:** 2015-04-30

**Authors:** Olga Kuksenok, Anna C. Balazs

**Affiliations:** 1 Chemical Engineering Department, University of Pittsburgh, Pittsburgh. PA 15261, USA

## Abstract

Human motion is enabled by the concerted expansion and contraction of interconnected muscles that are powered by inherent biochemical reactions. One of the challenges in the field of biomimicry is eliciting this form of motion from purely synthetic materials, which typically do not generate internalized reactions to drive mechanical action. Moreover, for practical applications, this bio-inspired motion must be readily controllable. Herein, we develop a computational model to design a new class of polymer gels where structural reconfigurations and internalized reactions are intimately linked to produce autonomous motion, which can be directed with light. These gels contain both spirobenzopyran (SP) chromophores and the ruthenium catalysts that drive the oscillatory Belousov-Zhabotinsky (BZ) reaction. Importantly, both the SP moieties and the BZ reaction are photosensitive. When these dual-functionalized gels are exposed to non-uniform illumination, the localized contraction of the gel (due to the SP moieties) in the presence of traveling chemical waves (due to the BZ reaction) leads to new forms of spontaneous, self-sustained movement, which cannot be achieved by either of the mono-functionalized networks.

Polymers containing both the SP and BZ functionalities have just recently been synthesized^1^. To the best of our knowledge, there have been no systematic studies of these dual-functionalized “SP-BZ” gels. Here, we uncover the properties of these active materials and provide guidelines for controlling the complex synergy between the structural reconfiguration and motion.

Before detailing the behavior of the SP-BZ gels, we first describe the distinctive properties of the mono-functionalized polymer networks. Notably, the SP-functionalized poly(*N*-isopropylacrylamide) (poly(NIPAAm)) gels^2^,^3^,^4^,^5^ can be molded remotely and reversibly into a variety of shapes *via* non-uniform illumination^6^. In acidic aqueous solutions and the absence of light, the spirobenzopyran chromophores remain primarily in the open-ring form (the protonated merocyanine form, or *McH*) and are hydrophilic^2^,^3^,^7^. Illumination with blue light causes the isomerization of these chromophores into the closed-ring form (the spiro form, or *SP*), which is hydrophobic^2^,^3^. Consequently, when this gel is illuminated through a photomask, the areas exposed to light shrink, while the non-exposed areas remain swollen. The resulting relief patterns can be “erased” simply by removing the light source^2^,^6^.

The remarkable feature of the BZ gels^8^,^9^ is that they are the only known polymer networks that undergo periodic swelling and deswelling in the absence of external stimuli and an imposed flow. (Other remarkable examples of oscillating gels include pH-responsive systems based on the bromate–sulfite (BS) reaction that undergo oscillations in a continuously-stirred tank reactor^10^ or gel-based oscillators that occur due to a chemo-mechanical feedback mechanisms in multi-component systems such as hydrogel-enzyme oscillators^11^,^12^,^13^ or self-regulating, homeostatic materials^14^.) When Ru(bpy)_3_ is grafted to a poly(NIPAAm) gel and the material is immersed in a solution of BZ reagents, the ensuing chemical reaction provides the fuel for this BZ gel's rhythmic mechanical oscillations. In particular, the gel swells as the Ru catalyst is oxidized and collapses when Ru is reduced by the oscillating reaction. These mechanical oscillations are accompanied by traveling chemicals that propagate throughout the sample (if the gel size exceeds the wavelength of the chemical wave). Millimeter-sized BZ gels can undergo self-oscillations for a few hours, i.e., until the reagents in the host solution are consumed^9^,^15^; the system, however, can be readily “re-fueled” by replenishing these solutes^16^.

The BZ functionality forms a perfect complement to the SP-functionalization because the BZ gels are also responsive to blue light. Non-uniform illumination with blue light can be harnessed to control the pattern of wave propagation and direct the motion of the BZ gels^17^,^18^,^19^. With both functionalities in one sample, the illuminated portion of the SP-BZ gel will shrink, altering the gel's overall shape. This, in turn, can drastically affect the dynamics of the wave propagation and the sample's motion, and thereby lead to completely new dynamic behavior.

The recently synthesized poly(NIPAAm-co-Ru(bpy)_3_-co-Sp) chains that encompass both the BZ and SP functionality are referred to as “PNRS”^1^. The SP-BZ gels can be formed by cross-linking PNRS chains^1^. Published experiments were, however, solely focused on probing the solution properties of the dissolved PNRS chains (i.e., no cross-linking)^1^. Importantly, these studies revealed that: 1) the chains exhibit an oscillatory coil-globule transition (due to the BZ reaction) and 2) the lower critical solution temperature (LCST) of the polymer solution can be shifted by light (due to the light-induced conversion of the chromophore to the hydrophobic form). These experiments indicate that the SP-BZ gels will integrate the distinctive properties provided by both the chromophores and the catalyst.

To explore potentially interesting behaviors of these new SP-BZ gels, we developed the computational model described in the *Methods*. (Unless noted otherwise, the simulation parameters and their correspondence to physical values are given in the SI.) In particular, we modified the gel lattice spring model, or gLSM^20^,^21^,^22^, which combines a finite element approach for solving the elastodynamic equations that characterize the propagation of chemo-mechanical waves in the gel, and a finite difference approximation for solving the terms that characterize the chemical reactions and diffusion of reactive species in the system. The gLSM was originally developed to model the dynamic behavior of BZ gels^8^,^23^,^24^,^25^,^26^, allowing us to capture the self-oscillation of the gel induced by the internalized BZ reaction. We build on this model to describe the SP-BZ gels; specifically, we now account for the kinetics of the photo-induced isomerization of the SP chromophores, and the corresponding decrease in the hydration of the polymer network caused by the isomerization of the chromophores to their spiro form. Moreover, we account for the effect of light on both the BZ reaction and SP moieties within the SP-BZ gels. The latter effect is introduced through two parameters: the reaction rate constant *k_L_*(*I*(**r**)), which describes the light-induced ring-closure of the spirobenzopyran moiety, and the coefficient Φ(*I*(**r**)), which accounts for the additional production of bromide ions in BZ reaction in the presence of light^27^. Both parameters are assumed to be proportional to the light intensity at a given point, *I*(**r**). In accordance with experimental findings^2^, we also assume that the temperature of the SP-functionalized gels remains constant when they are illuminated with blue light. Here, we investigate the dynamic behavior of the dual-functionalized gels in the presence of non-uniform light, focusing on the illumination patterns described below. In addition, we elucidate the effects of varying the total concentration of the spirobenzopyran chromophores, 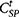, on the directed motion of these systems.

The output from the simulations in Fig. 1 illustrates the dynamic behavior in these dually responsive gels. Here, an initially flat sample roughly 6.5 *mm* × 1.7 *mm* × 0.2 *mm* in size is illuminated at both edges, leaving a central non-illuminated region of radius *R_d_* = 20 units (approximately 0.8 *mm*). The sample morphs into a bent structure that promotes its self-sustained downward motion. Neither the SP nor the BZ gel alone would yield such net translational motion (see Supplementary Information).

The SP-BZ gel encompasses two important properties arising from the BZ functionalization that contribute to the above behavior. First, due to the inter-diffusion of the polymer and solvent in the system, BZ gels autonomously move in a direction that is opposite to the direction of the traveling chemical wave within the material^17^,^18^,^19^. Second, light of a sufficiently high intensity suppresses oscillations within an illuminated region, causing the wave propagation to originate in the non-illuminated area of the BZ gel^17^,^18^,^19^.

In Fig. 1, the intensity of the light illuminating the ends is higher than the critical intensity needed to suppress the oscillations in a uniformly illuminated sample of the BZ gel. (In eq. (11) below, we set Φ = 4 × 10^−4^ > Φ_c_ within the illuminated regions.) Hence, the chemical waves originate only in the dark central region and then propagate to the illuminated ends. Due to the spirobenzopyran chromophores, the illuminated ends of the gel shrink. Compared to these collapsed ends, the central region is relatively swollen. This uneven distribution of solvent within the gel causes the central region to bulge out of the plane (in the *negative* *z*-direction for the case in Fig. 1). With the bending of the gel, the traveling chemical waves move not only in the lateral direction (the *x-*direction), but also upward from the depressed center to the ends of the sample (along the *positive z-*direction). (The pattern development during a single oscillation cycle is shown in Figure S1 a–c.) Due to the inter-diffusion of the polymer and solvent^20^,^21^,^28^, the movement of the chemical wave in the positive *z*-direction causes the gel to move in the opposite direction^17^,^18^,^19^; i.e., the *negative* z-direction, and hence, migrate to the bottom of the simulation box for the example in Fig. 1. (The differences in the chemical patterns among Figs. 1a–c are due to the fact that these images are taken at different phases of the chemical oscillations.)

Since we neglect the attenuation of light in our relatively thin samples, the “upward” and “downward” directions of motion occur with equal probability in our simulations (Supplementary Fig. S1, d). If the sample is illuminated from the top, even a small degree of attenuation would break this symmetry and favor the downward bending (due to the higher degree of shrinking on the illuminated top of the sample) and would result in the downward motion shown in Fig. 1.

To characterize the observed motion, we monitor the temporal evolution of the *z*-coordinate of the central node on the bottom face of the gel, *z*_c_ for different values of 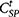 (Fig. 2). In all cases, the system's dynamics can be divided into two stages. During the first stage, the sample remains flat and localized in one plane (as indicated by the flat portion of the curve). During the second stage, the sample moves downward (along the negative *z*-direction) with approximately constant velocity. (The letters within the curve for 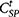 = 0.2 in Fig. 2a correspond to the respective images in Fig. 1.) The seemingly monotonic movement of the sample involves small-scale oscillatory motion, as can be seen from the enlargement of a portion of the curve (Fig. 2a).

Figure 2b reveals two key effects of varying 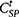, indicating means of controlling the autonomous motion. First, the duration of the first stage is the shortest for the highest concentration of 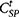 (see inset). During this first stage, the out-of-plane bending occurs due to the mismatch in the degree of swelling of the central region and illuminated, shrunken ends. The ends shrink significantly more at higher 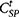 (Supplementary Fig. S2 showing the equilibrium degree of swelling of as a function of 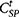); this results in a higher mismatch between the dark and illuminated regions, and correspondingly, a faster loss in the stability of the in-plane configuration. This behavior can be seen by comparing the early-time images for samples with low and high values of 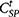 (Fig. 3a and c, respectively).

Second, the velocity of the downward motion is fastest for the lowest 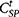 and decreases with an increase in 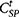. Notably, the degree of bending also depends on 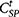 and is lower for the higher 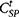 due to the lower degree of swelling (Supplementary Fig. S2) and higher rigidity of the sample; namely, the presence of additional bulky chromophores makes the sample less flexible at higher 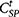. Hence, the vertical components of the chemical wave propagating from the center to the ends of the gel are significantly more pronounced for the lower 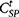 sample, which exhibits a higher degree of bending (see late-time images in Fig. 3b and d). The latter behavior leads to the faster downward motion at lower 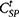.

We emphasize that both types of functionalization are necessary for a flat, thin gel to undergo structural reconfiguration (bending) that leads to autonomous, directed motion. A pure SP gel would simply bend, but not move under a stationary light^6^. If the pure BZ gels were placed in the non-uniform illumination considered here, the sample would undergo spontaneous movement that depends on initial random fluctuations in the concentration of BZ reagents (Supplementary Figs. S3 and S4). In the dual-functionalized gel, the SP chromophores give rise to the controllable out-of plane bending, which modulates the propagation of the traveling BZ chemical waves. These synergistic interactions enable the self-sustained motion shown in Fig. 1.

Our simulations reveal that the light-directed shape-changes and resultant motion are independent of initial fluctuations (Supplementary Fig. S1). (We ran four independent simulations for all the values of 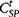 in Fig. 2b and in all cases observed the robust bending and directed motion of the sample displayed in Figs. 1–3.). Additional simulations show that such behavior remains robust for a range of radii of the dark region, 8 < *R_d_* ≤ 25. If, however, we increase *R_d_* beyond 25 units, and thereby decrease the size of the ends that undergo light-induced shrinking, we find that the SP-BZ gel undergoes complex motion (Fig. 4), which depends on initial random fluctuations and is similar to that of pure BZ gels (see Figs. S3 and S4). In Fig. 4, where *R_d_* = 30, the sample effectively reorients to “escape” from the illuminated areas and moves towards the dark region in the center. The latter behavior is similar to the response of pure BZ gels, which exhibit negative photo-taxis^17^,^18^,^19^,^29^,^30^. These results indicate that the illuminated region must be above a critical size to drive the gel to undergo the robust out-of-plane bending (and subsequent directed motion).

To demonstrate the generality of behavior seen in Fig. 1, we considered a relatively large, square sample that is 90 × 90 × 5 nodes in size and illuminated on all four edges, leaving the central radius of *R_d_* = 20 in the dark (Supplementary Fig. S5). Again, the out-of-plane bulging of the swollen center is accompanied by the bending of the collapsed edges, resulting in the directed motion of the gel. For the case in Supplementary Fig. S5, all four corners are seen to fold upward and the gel moves downwards. Decreasing the size of the illuminated area so that *R_d_* = 40 eliminates this effect and the sample displays behavior (Supplementary Fig. S6) analogous to that in Fig. 4.

Besides the size of the illuminated region, the actual pattern of illumination also affects the systems' dynamics. When the light and dark regions for both the rectangular (Fig. 1) and square-shaped samples (Supplementary Fig. S5) are reversed (so that the center is illuminated and the ends are held in the dark), the more swollen regions are located at the edges of the gels (Supplementary Fig. S7). Hence, the samples lack a central “focal” point (bent region) that leads to the directed motion; hence, the resulting motion in these cases strongly depends on initial fluctuations (Supplementary Fig. S7).

While certain modes of non-uniform illumination can be harnessed to drive the gel's directed motion, uniform illumination over the entire sample can be exploited to arrest the motion. At this light intensity (Φ > Φ*_c_*), the oscillations in the BZ gel would be suppressed. Moreover, under the uniform illumination, the entire sample would exhibit the same equilibrium degree of swelling, and hence, it would straighten out to its initial, flat state. The prior motion can, however, be reinstated by illuminating just the ends of the gel.

With this newly derived model, we could probe the properties of a new material, which permits remarkable forms of controllable, biomimetic movement. Namely, the photo-induced, coordinated contraction and expansion of the gel is coupled to the chemo-mechanical transduction (enabled by the internalized reaction) to produce self-sustained motion. The process of structural reconfiguration leading to net movement resembles the action in muscle-driven motion. Notably, the BZ functionality allows these materials to move autonomously, without the need for external stimuli. In effect, the system generates the energy to power its motion. The SP-functionality allows one to not only reshape and bend the material, but also capitalize on the photosensitivity of the BZ gel and thus, modulate the path of the traveling chemical waves in the material. Hence, with the combination of light and the dual-functionalization, the gel's shape, movement and directionality can be altered “on the fly”. In essence, this light-responsive material offers a blank canvass for molding self-propelled, reconfigurable soft robots that could operate in an autonomous manner.

## Methods

To perform these studies, we modified our gel lattice spring model, or gLSM^20^,^21^,^22^, which was originally developed to capture the dynamic behavior of BZ gels^8^,^23^,^24^,^25^,^26^. The total energy of these responsive gels is the sum of the interaction energy, *U_FH_*, and the elastic energy associated with the deformation of the gel, *U_el_*. The interaction energy is given as: 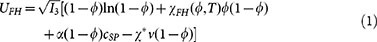
The first two terms in eq. (1) describe the mixing energy of the system. Here, *χ*_FH_(*φ*,*T*) is the polymer-solvent interaction parameter^31^, which depends on the polymer volume fraction, *φ*, and temperature, *T*. The last two terms in eq. (1) describe changes in the hydration of the gel due to interactions involving the chemically anchored moieties. The parameters *α* and χ* in eq. (1) are the coupling parameters that characterize the strengths of the respective interactions. By setting *α* > 0, we can account for the photo-induced shrinking of the gel^6^ caused by the photo-conversion of the chromophores to the spiro (SP) form^2^,^3^. Here, c*_SP_* is the concentration of chromophores in the SP state normalized by the total chromophore concentration, 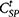. By setting χ* > 0, we can account for relative swelling of the gel due to the hydrating effect of the oxidized ruthenium catalyst on the network^21^. The concentration of oxidized ruthenium catalyst in the system is given by *v*. The factor *I*_3_ in front of eq. (1) is given by 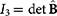 and is an invariant of the left Cauchy-Green (Finger) strain tensor 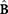; it characterizes the volumetric changes in the deformed gel^32^. The local volume fractions of polymer in the deformed state, *φ*, and undeformed state, *φ*_0_, are related as Ref. 21 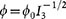.

The elastic energy contribution, *U_el_*, to the total energy describes the rubber elasticity of the cross-linked network^33^,^34^. It is proportional to the crosslink density, *c*_0_, as follows:
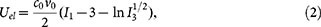
where *v*_0_ is the volume of a monomeric unit and 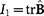 32.

Equations (1) and (2) yield the following constitutive equation for SP-BZ gels^20^,^21^:
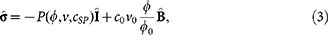
where 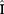 is the unit tensor, 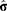 is the dimensionless stress tensor measured in units of 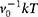. The isotropic pressure in eq. (3) depends on both the concentration of oxidized ruthenium catalyst, *v*, and concentration of chromophores in the spiro form, c*_SP_*, and can be written as:
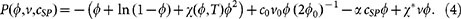
Here, *χ*(*φ*,*T*) = *χ*_0_(*T*) + *χ*_1_*φ*, where χ_0_*(T)* = [δ*h* − *T*δ*s*]/*kT*, with δ*h* and δ*s* being the respective changes in the enthalpy and entropy per monomeric unit of the gel^31^.

We describe the dynamics of these gels within the framework of the two-fluid model^34^, where both the respective polymer and solvent velocities, **v**^(*p*)^ and **v**^(*s*)^, contribute to the total velocity of the system as **v** = *φ***v**^(*p*)^ + (1−*φ*)**v**^(*s*)^. We further assume that only the polymer-solvent inter-diffusion contributes to the gel dynamics^20^,^21^,^28^; therefore, we set ***v*** = 0 20,21.

The above equations for the elastodynamics of the gel must be supplemented by kinetic equations for the chromophores, which undergo the following inter-conversion:
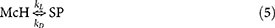
where *k_L_* and *k_D_* are the reaction rate constants for the forward and backward reaction, respectively. Typically, spontaneous conversion back to the *McH* form is significantly slower than the photo-induced isomerization to the SP form^3^,^7^. This inter-conversion reaction can be described as Ref. 6:
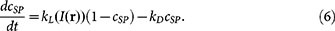
The reaction rate constant *k_L_* is proportional to the local light intensity, *I*(**r**). We consider only thin samples, and thus, neglect the attenuation of the light across the thickness of the sample^6^. The photo-stationary concentration of chromophores in the SP form yields^6^ 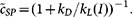


We must also account for the reaction kinetics in BZ gels. This can be captured by a modified version^20^,^21^ of the two-variable Oregonator model^35^,^36^ that explicitly accounts for the polymer volume fraction, *φ*^20^,^21^.

As noted above, the polymer and solvent move with the respective velocities of **v**^(*p*)^ and **v**^(*s*)^. Hence, the chemical reactions within the SP-BZ gels occur simultaneously with the following three dynamic processes: 1) the movement of the grafted oxidized catalyst, *v*, and chromophores, c*_SP_*, with the polymer at the velocity **v**^(*p*)^, 2) the transport of the activator for the BZ reaction, *u*, along with the solvent at the velocity **v**^(*s*)^ = −*φ*/(1−*φ*)**v**^(*p*)^, and 3) the diffusion of the dissolved activator *u* throughout the polymer network with a diffusion flux given by^21^ **j**^(*u*)^ = −(1−*φ*)∇(*u*(1−*φ*)^−1^). Hence, the dynamics of the SP-BZ gels can be described by the following set of equations:
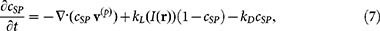

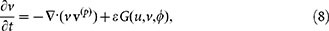

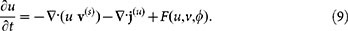
The terms *G*(*u*,*v*,*φ*) and *F*(*u*,*v*,*φ*) in eqs. (8)–(9) are the reactive terms that characterize the BZ reaction and are written as:
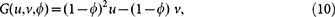

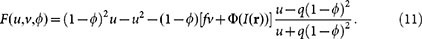
The dimensionless parameters *q*, *f*, and *ε* in the above equations have the same meaning as in the original Oregonator model^35^. The dimensionless variable Φ(*I*(**r**)) in eq. (11) accounts for the additional production of bromide ions in the presence of light^27^ and is dependent on the light intensity *I*. In our simulations of BZ gels, the above approach allowed us to reproduce^17^,^18^ the experimentally observed suppression of oscillations within BZ gels by visible light above a critical intensity^25^.

Within the framework of the gLSM model^20^,^21^,^37^, the dynamics of the polymer network is assumed to be purely relaxational^38^, so that the forces acting on the deformed gel are balanced by the frictional drag due to the motion of the solvent, resulting in the following force balance equation^21^:
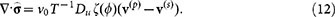
Here, *ζ*(*φ*) is the friction coefficient, *D_u_* is the diffusion coefficient of the activator, and 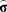 is defined above (eq. (3)). We assume^38^ that the gel/solvent system satisfies the incompressibility condition, ∇⋅**v** = 0; in addition, we set the total velocity, **v** ≡ *φ* **v**^(*p*)^ + (1−*φ*)**v**^(*s*)^ = 0 21. In other words, it is solely the polymer-solvent inter-diffusion that contributes to the gel dynamics^21^,^38^ and, correspondingly, there is no net momentum exchange between the gel and the external fluid. Hence, by setting **v** = 0 21,38, we neglect the hydrodynamic interactions within the gels. In fact, neutral, non-responsive polymer gels were utilized as a medium for the BZ reaction to specifically suppress the hydrodynamics effects^39^. In a previous study, we showed that we can neglect hydrodynamic effects even for the case of multiple, interacting gels due to the slow dynamics of the gels and the low viscous forces in the system^40^.

The effect of light in our SP-BZ gels is introduced through the reaction rate constant *k_L_*(*I*(**r**)) in eq. (7) and the coefficient Φ(*I*(**r**)) in eq. (11); both values are assumed to be proportional to the light intensity at a given point, *I*(**r**). Notably, the temperature of the SP-functionalized gels remains constant when it is illuminated with blue light^2^, and hence, it is only the light-induced ring closure that causes the dehydration of the polymer matrix. In other words, the physical origin of the photo-induced volume change of the SP-functionalized gels is distinctly different from gel collapse arising from direct light-induced heating.

## Supplementary Material

Supplementary Information Supplementary Information

## Figures and Tables

**Figure 1:**
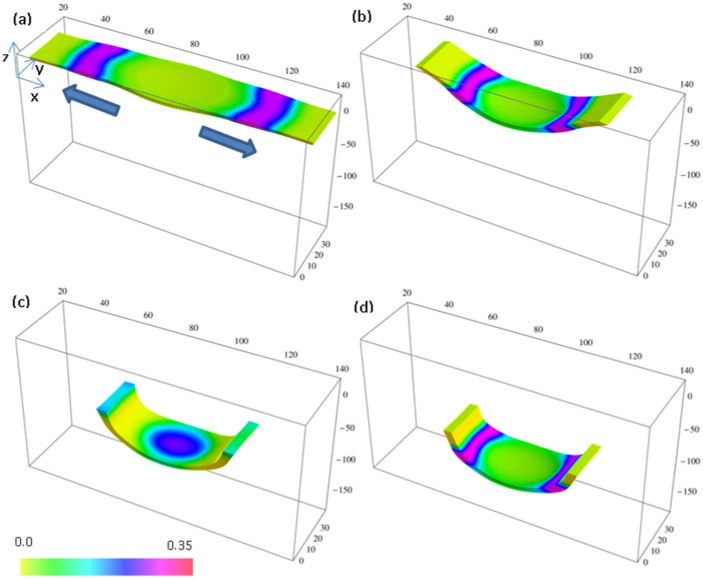
Dynamics of a SP-BZ gel with total concentration of chromophores 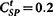. Simulation times are: (a) *t* = 29088, (b) *t* = 6 × 10^4^, (c) *t* = 1.4 × 10^5^ and (d) *t* = 1.8 × 10^5^. Both ends of the sample are illuminated; radius of the masked (non-illuminated) region in the center is R_d_ = 20 units (approximately 0.8 *mm*). The detailed correspondence between the dimensionless simulation values and physical values are given in the SI; the dimensionless units of time and length in our simulations correspond to ~1 sec and ~40 *μm*, respectively. Color bar indicates the concentration of oxidized ruthenium catalyst, *v*.

**Figure 2:**
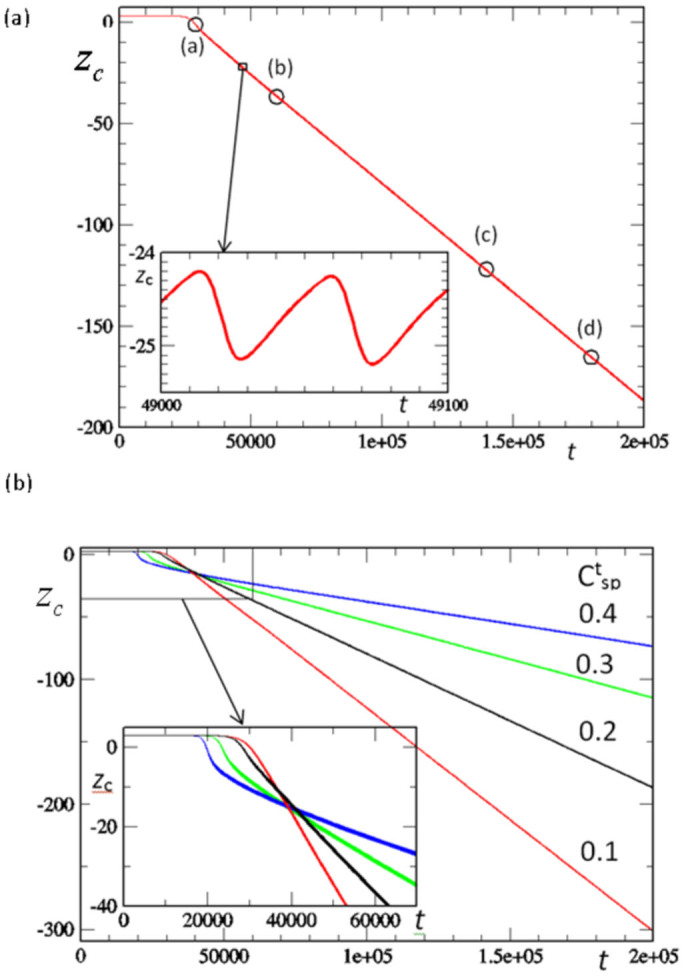
(a) Time evolution of the *z*-coordinate of the bottom face of the center of the gel, *z*_c_, for the simulation in Figure 1. Points marked (a)–(d) correspond to the respective images in Fig. 1 a–d. (b) Time evolution of *z*_c_ for gels at four different values of 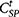. Inset shows evolution at early times.

**Figure 3:**
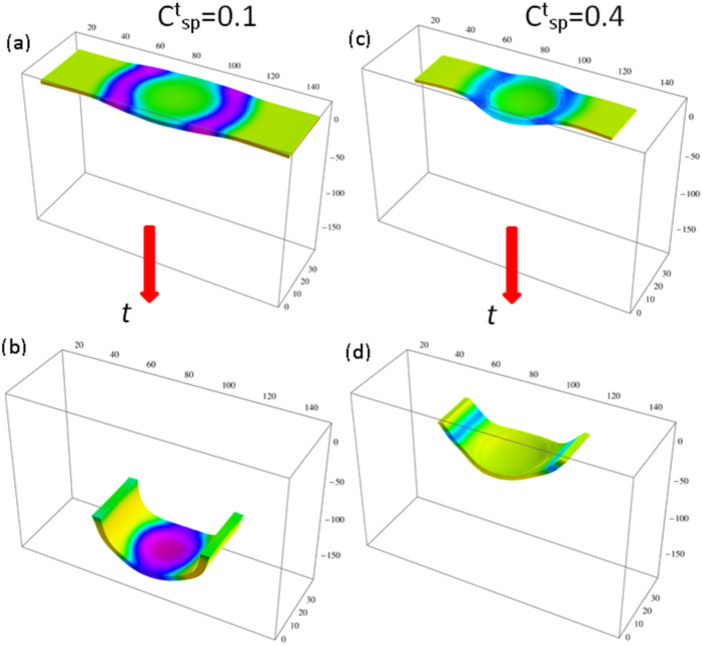
Dynamics of a SP-BZ gel at low concentration of chromophores, 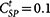 (left column) and high concentration of chromophores, 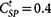 (right column). Simulation times are: (a) *t* = 29056 and (b) *t* = 1.3 × 10^5^ for 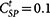, and (c) *t* = 29044 and (d) *t* = 1.3 × 10^5^ for 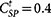.

**Figure 4:**
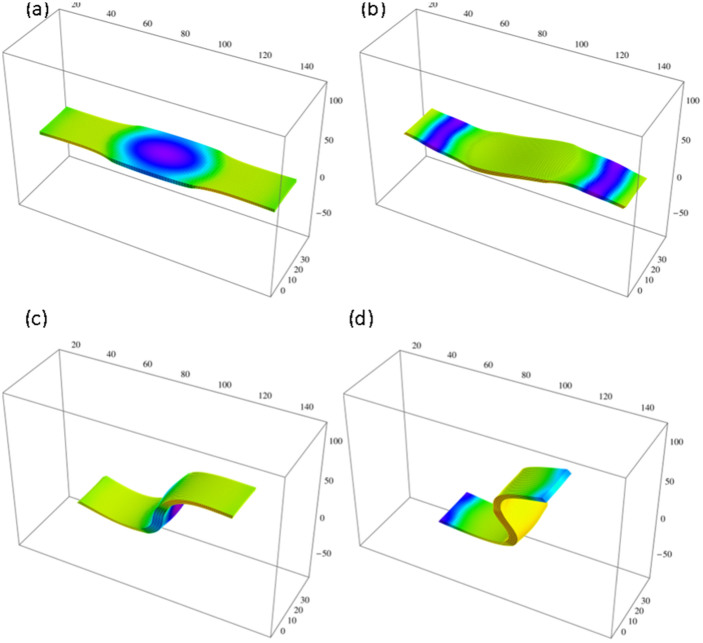
Dynamics of a SP-BZ gel with 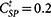. Simulation times are: (a) *t* = 4 × 10^4^, (b) *t* = 9 × 10^4^, (c) *t* = 1.3 × 10^5^ and (d) *t* = 1.5 × 10^5^. Both ends of the sample are illuminated; radius of the non-illuminated region in the center is R_d_ = 40.
