# Supplementary material for: Designing Dual-functionalized Gels for Self-reconfiguration and Autonomous Motion
Source: Sci Rep. 2015 Apr 30;5:9569. doi: 10.1038/srep09569 (PMC5386209; doi:10.1038/srep09569)
Supplement: Supplementary Information [file srep09569-s1.pdf]

Designing Dual-functionalized Gels for Self-reconfiguration and Autonomous Motion

Olga Kuksenok and Anna C. Balazs*

Chemical Engineering Department, University of Pittsburgh

Pittsburgh, PA 15261, USA

E-mail: balazs@pitt.edu

Supplementary Information

A. Simulation Parameters and their Relationships to the Experimental Values

For a set of reference parameters, we chose the values described below, which are based on the experimental data available for pure BZ gels [1, 2] , pure spirobenzopyran-functionalized gels [3, 4], and the dual-functionalized polymer chains (the so-called PNRS, or poly(NIPAAm-co-Ru((bpy)$_3$-co-Sp) [5]. For the BZ reaction parameters, we set [6] $\varepsilon = 0.354$ and $q = 9.52\times10^{-5}$ and for the parameters characterizing the properties of the gel, we set $\phi_0 = 0.139$, and $c_0 = 1.3\cdot10^{-3}$. For the polymer-solvent interaction parameters, we used $\chi_0 = 0.338$ and $\chi_1 = 0.518$; these values correspond to a temperature of 20^0C for a gel with the above ϕ_0 and c_0, using the temperature dependence of χ_0 for the poly(NIPAAm) gels given by S. Hirotsu[7]. We also set $f = 0.8$ and $\chi^* = 0.05$; χ^* is an adjustable parameter of the model that accounts for the hydrating effect of the oxidized catalysts on the BZ gels [6] . The choice of these parameters sets the dimensionless units of time and length in our simulations to be $\sim 1\sec$ and $\sim 40\mu m$, respectively [6].

We now turn to the parameters that account for the functionalization with spirobenzopyran chromophores in our dual-functionalized gels. For the reference values of the forward and backward reaction rates, we set $k_L = 10^{-3}$ and $k_D = 5 \cdot 10^{-5}$. With the scaling for dimensionless units of time provided above, these values correspond to the dimensional values of $\sim 10^{-3} s^{-1}$ and $5 \cdot 10^{-5} s^{-1}$ for the respective rate constants for photo-induced ring closure and spontaneous ring opening in the dark. With these reaction rate constants, photoisomerization during one hour results in a conversion of about 92% of chromophores into the spiro form, while spontaneous ring opening in the dark will take about 20 hours. These values agree well with the corresponding experimental values [5] for PNRS polymers with dual functionalization. In addition, with the ratio $k_D / k_L = 20$, the photo-stationary concentration of chromophores in the spiro form is $\tilde{c}_{SP} \approx 0.95$. This value is within the range provided in refs. 3, 4, 8, where researchers showed that isomerization from the *McH* to the *SP* form is typically within the range from 82% to 98% in the photo-stationary state, depending on the specific spirobenzopyran derivatives that were used.

We set the interaction parameter that describes the photo-induced decrease in the hydration of the polymer network to[9] $\alpha = 8.25 \times 10^{-2}$; this parameter is the only fitting parameter in the model for the pure spirobenzopyran-functionalized gels. This parameter was chosen to produce a light-induced decrease in the gel's degree of swelling [9] that is similar in magnitude to the corresponding experimental values [3, 4].

As initial conditions for the simulations below, we chose the concentrations of the oxidized catalyst, v, and activator, u, to be randomly distributed around their stationary solutions, v_{st} and u_{st}, in the absence of light [10]. The size of each element is taken to be a cube with side λ_{st} defined by the value of the stationary solution for the polymer volume fraction, ϕ_{st} [10]. These parameters are

set as: $v_{st} = 0.1755$, $u_{st} = 0.1836$, and $\lambda_{st} = 1.470$. In the simulations described below, the reference sample size is $110 \times 30 \times 5$ elements, which corresponds to the dimensional size of $6.5mm \times 1.7mm \times 0.2mm$ in the absence of illumination.

B. Additional Simulation Results

Below, we provide additional plots that are referred to in the main text of the manuscript.

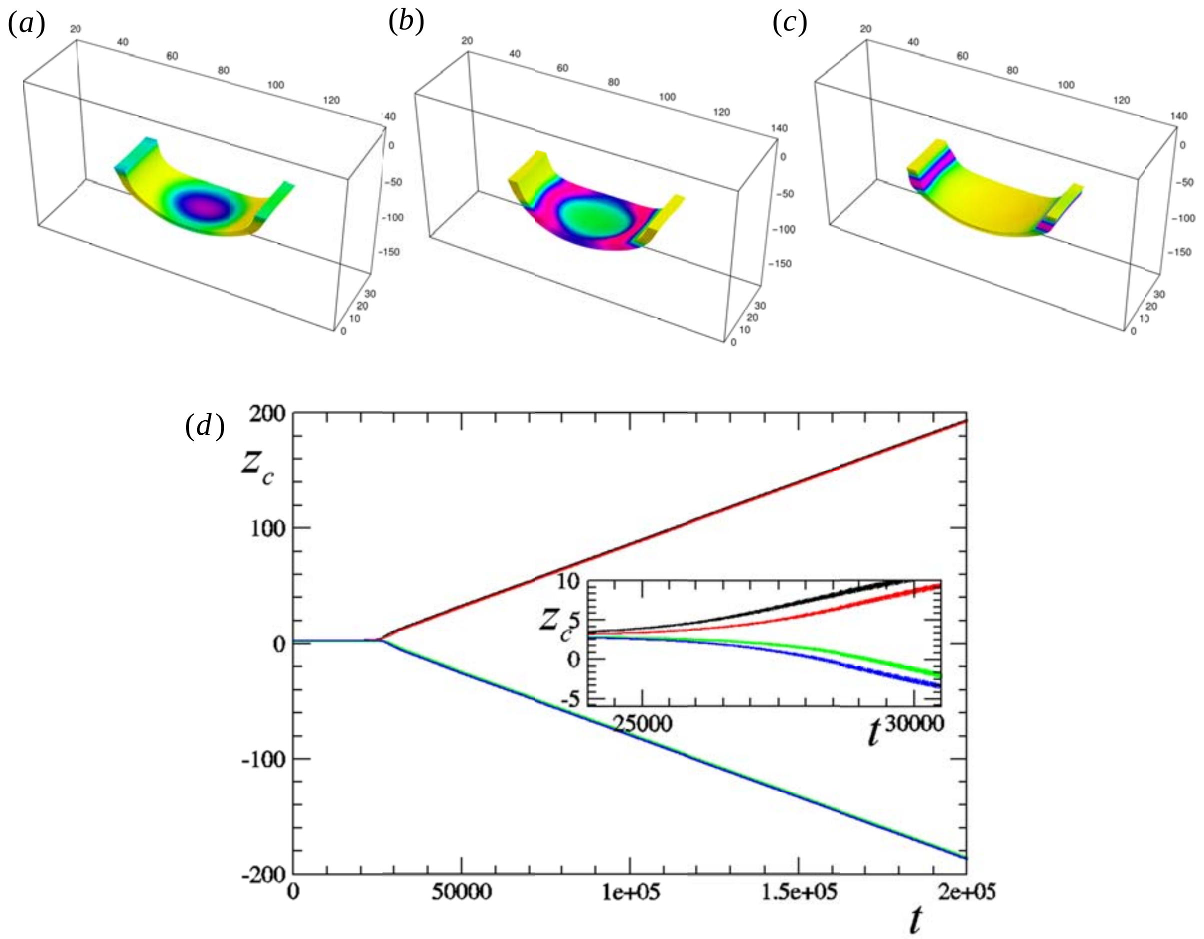

Figure S1. (a-c) Late time dynamics of a SP-BZ gel with total concentration of chromophores $C_{SP}^{t} = 0.2$ during a single oscillation period for the same run as shown in Figure 1. Simulation times are: (a) *t*=149032, (b)149044, (c) *t*=149060. These images show that the chemical wave is initiated

at the center of the sample and propagates towards its ends during the single oscillation period, thereby creating a vertical component (“up”) of the chemical wave propagation. Due to the inter-diffusion of polymer and solvent, the gel moves in the direction opposite to that of wave propagation [11, 12, 13], i.e., it moves down in this simulation example. (d) Time evolution of the z-coordinate of the center of an SP-BZ gel, z_c, for four independent simulations. The sample is illuminated at both ends and the radius of the central, masked region is $R_d = 20$. Here, $C_{SP}^t = 0.2$. The structural reconfiguration and motion of the gel for one of these simulations is shown in Fig. 1 of the main text. Inset shows the dynamics of the samples at the onset of the out-of-plane motion. While the “up” and “down” motion occur with equal probability, the directed motion in the vertical direction is robust and does not depend on initial random fluctuation (unlike the case of pure BZ gels, as discussed below). We also confirmed that this robust motion in the vertical direction does not depend on the initial random seed used in the simulation when we varied the concentration of spirobenzopyran chromophores (namely, for $C_{SP}^t = 0.1$, $C_{SP}^t = 0.3$, and $C_{SP}^t = 0.4$).

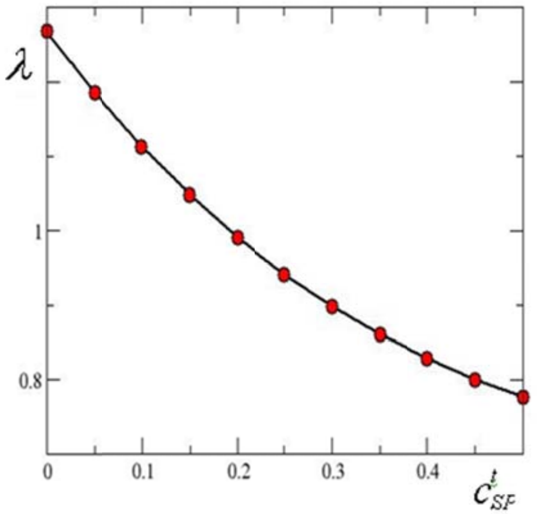

Figure S2. Dependence of the equilibrium degree of swelling, λ, on the total concentration of spirobenzopyran chromophores, C_{SP}^t, for the sample of size $5 \times 5 \times 5$, under uniform illumination with $k_L = 10^{-3}$.

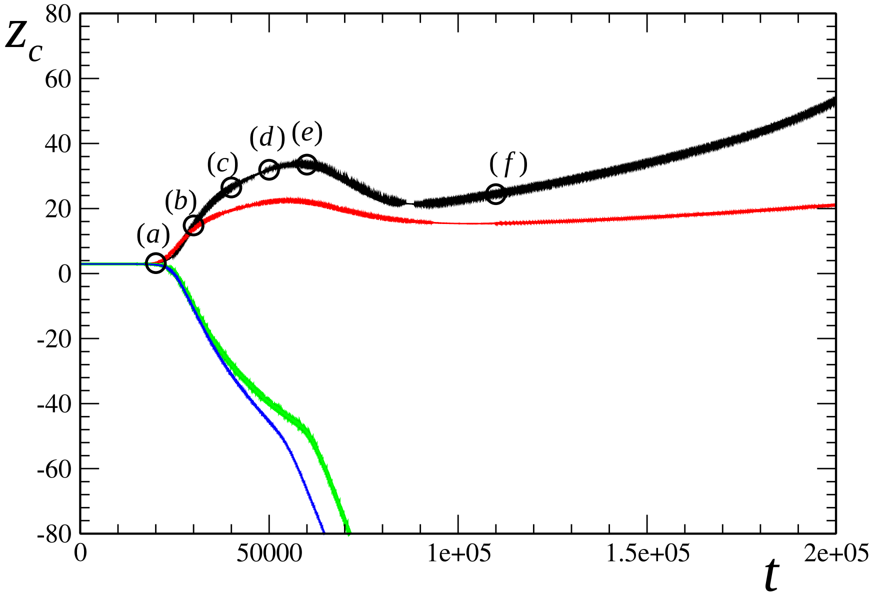

Figure S3. Time evolution of z_c, the *z*-coordinate of center of the pure BZ gel ($C_{SP}^t = 0$), for four independent simulations. Except for the value of C_{SP}^t, the rest of the parameters are the same as in Fig. 1 of the main text. Both ends of the sample are illuminated; the radius of the central, masked region is $R_d = 20$. Snapshots showing the evolution of a sample for one of these cases are given in Figure S4 below. (The images (a)-(f) in Figure S4 correspond to the instances in time marked with the circles (a)-(f) on the black line.) Another possible outcome for the evolution of the pure BZ gels is a train of chemo-mechanical waves propagating throughout the sample; this scenario again results in the random, uncontrollable motion of the sample. Our simulations show that the patterns of wave propagation that develop in this system strongly depend on initial conditions; hence, the resulting motion of the BZ gel also depends on initial fluctuations in the concentration of oxidized catalyst v,and activator, u. Thus, controlling the mode of motion requires the dual functionalization.

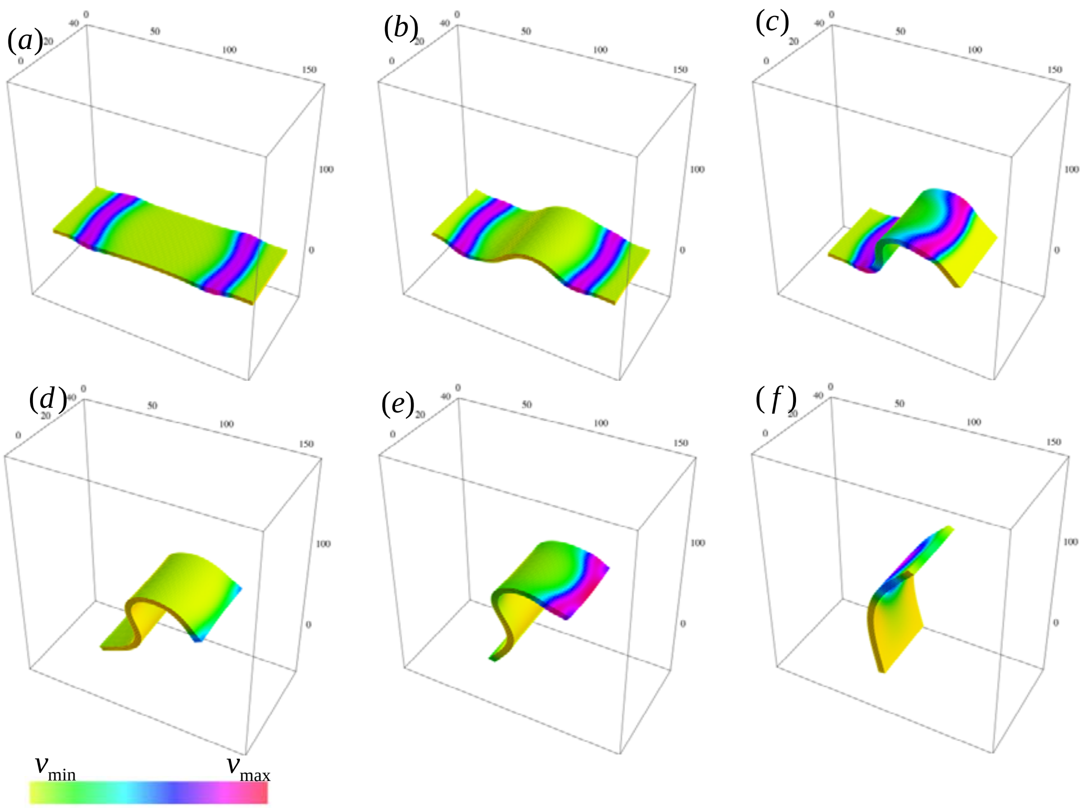

Figure S4. Evolution of the pure BZ gel in non-uniform light. Both ends of the sample are illuminated; the radius of the masked region in the center is $R_d = 20$. The times are as follows: (a) $t = 2\times10^4$, (b) $t = 3\times10^4$, (c) $t = 4\times10^4$, (d) $t = 5\times10^4$, (e) $t = 6\times10^4$, (f) $t = 1.1\times10^5$. The evolution of the z-coordinate of center of the gel is given by the black line in Figure S3.

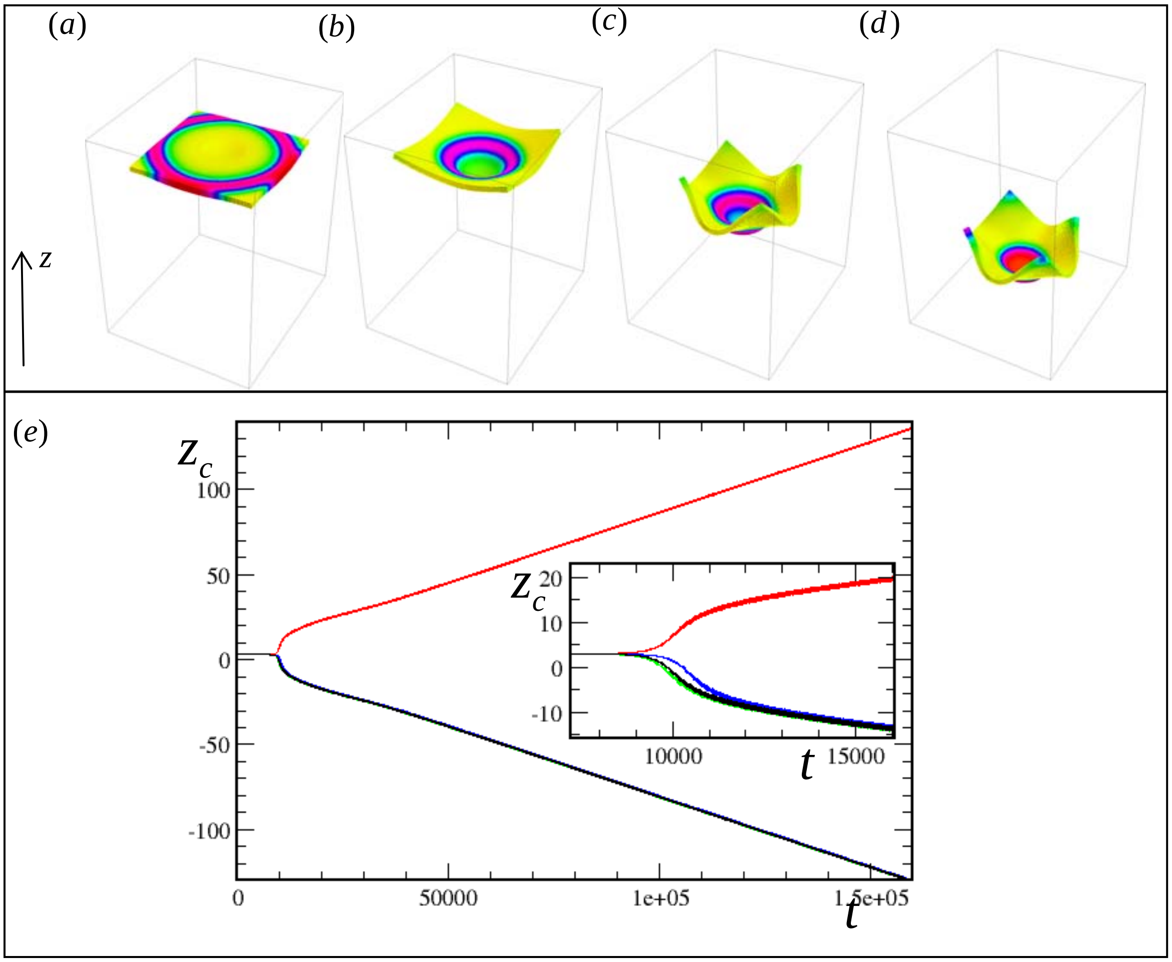

Figure S5. (a-d) Evolution of an SP-BZ gel of size 90x90x5 in non-uniform light. The radius of the central masked region $R_d = 20$; the rest of the sample is illuminated. Here, $C_{SP}^{t} = 0.2$. In this case, the ends and all the edges of the sample undergo light-induced shrinking, which in turn causes the "folding" of the gel. The times are as follows: (a) $t = 10^4$, (b) $t = 3\times10^4$, (c) $t = 9\times10^4$, and (d) $t = 1.5\times10^5$. (e) Time evolution of the z-coordinate of the center of the SP-BZ gel in the non-uniform light for four independent simulations. The inset shows the dynamics of the samples at the onset of the out-of-plane motion. Similar to the case in Fig. 1 of the main text and Fig. S1, the

vertical motion is robust and does not depend on random fluctuations. (As noted above, the "up" and "down" motions are equivalent in our simulations since we do not account for the light attenuation.) For the same sample size, we also found that the vertical motion remains robust and does not depend on the initial random seed used in the simulation when we varied the total concentration of spirobenzopyran chromophores (namely, for $C_{SP}^{t} = 0.1$, $C_{SP}^{t} = 0.3$, and $C_{SP}^{t} = 0.4$). Finally, we also found that for the case of pure BZ gels, the motion of the sample of the same size strongly depends on initial random fluctuations, similar to the case shown in Figs. S3-S4.

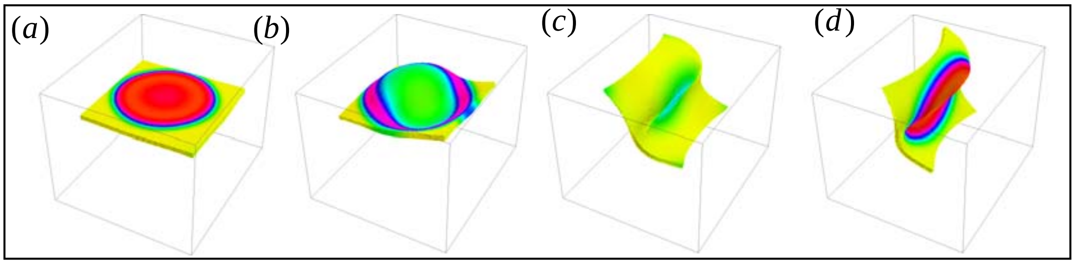

Figure S6. (a-d) Evolution of the SP-BZ gel of size 90x90x5 in non-uniform light. The radius of the masked region in the center is $R_d = 40$; the rest of the sample is illuminated. Similar to the case involving the rectangular sample (see Fig. 4), increasing the radius of the masked region (and thereby decreasing the size of the edges that undergo light-induced shrinking) produces dynamical behavior that depends on initial fluctuations. As in Fig. 4, the sample reorients to fit into the dark region. The times are as follows: (a) $t = 10^4$, (b) $t = 2 \times 10^4$, (c) $t = 7 \times 10^4$, and (d) $t = 9 \times 10^4$. To summarize, similar to the observed behavior for the rectangular samples, the directional motion is robust for the smaller sizes of R_d; however, increases in R_d cause the motion to be dependent on initial random fluctuations, displaying a pronounced tendency to turn towards the dark region (in the center of the simulation box).

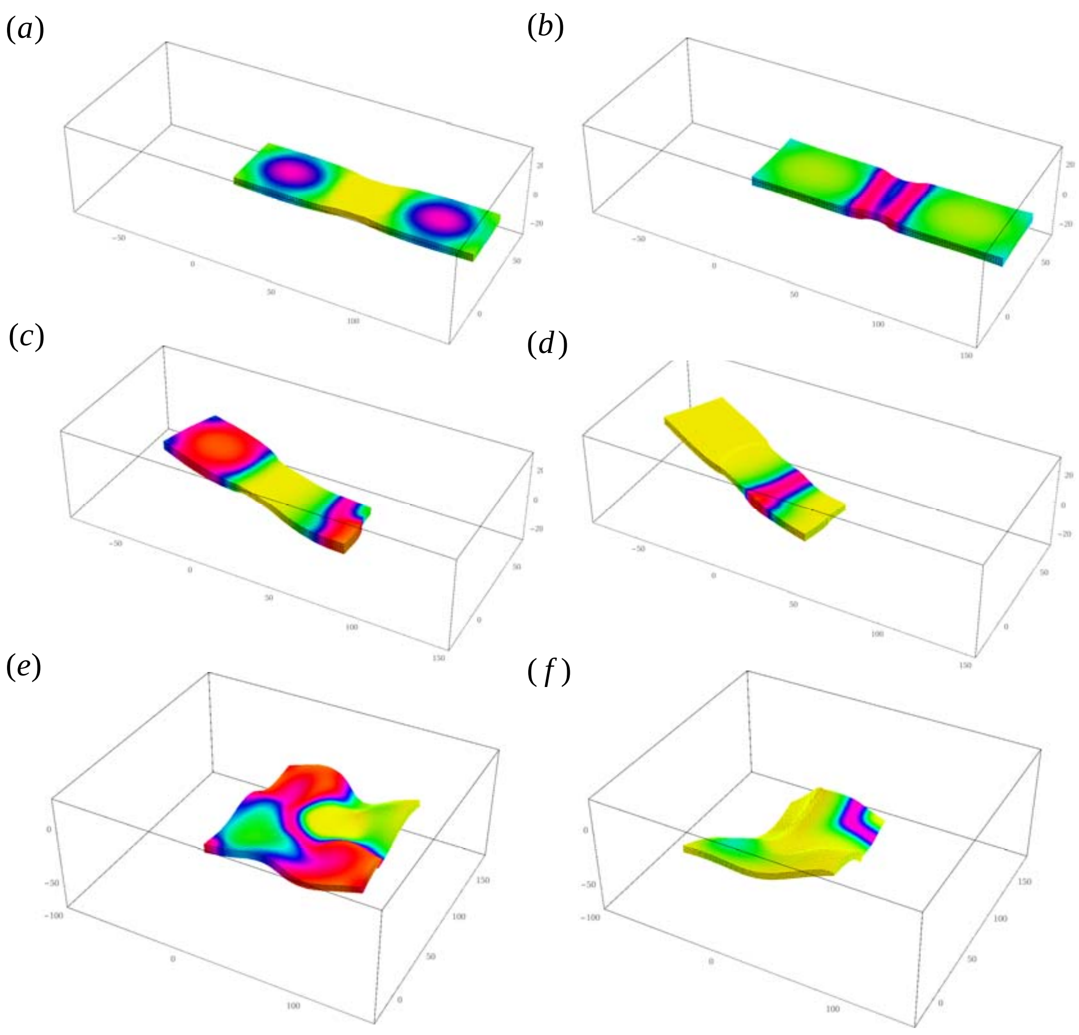

Figure S7 (a-d) Evolution of an SP-BZ gel of size 110x30x5 under non-uniform illumination. Here, the pattern of illumination is reversed with respect to that in Fig. 1 of the main text. The radius of the illuminated region in the center is $R_L = 20$ and the rest of the sample is in the dark. The simulation times are as follows: (a) $t = 9{,}036$, (b) $t = 9{,}052$, (c) $t = 10^5$, and (d) $t = 1.3 \times 10^5$. At early times, the sample remains in-plane, with the chemical waves originating from both ends of the sample and propagating towards the center (see (a-b)). At later times, due to initial random fluctuations, the wave propagation is no longer symmetric with respect to the center of the sample (see c-d), and the sample undergoes net motion in the direction opposite to that of wave propagation. While the gel in this example moved predominantly to the left, this direction of motion

is random and depends on the initial random seed used in the simulation. Ultimately, the sample moves away from the illuminated region. We note that the relatively shrunken region in the middle of the sample in (c) and (d) is still observed despite this portion of the sample being out of the illuminated region due to the low reaction rate coefficient for the spontaneous conversion of the chromophores back into their open-ring form in the dark. (e-f) Evolution of an SP-BZ gel of size 90x90x5 in the non-uniform light. The illumination pattern is reversed with respect to that in Fig. S5. Similar to the case in (a-d), at early times, the waves originate within the dark outer edges of the sample and at later times, the sample moves out of the illuminated region. The simulation times are $t = 2 \cdot 10^4$ in (e) and $t = 10^5$ in (f).

References

1. Yoshida, R., Kokufuta, E., Yamaguchi, T. Beating polymer gels coupled with a nonlinear chemical reaction. *Chaos* **9,** 260-266 (1999).
2. Sasaki, S., Koga, S., Yoshida, R., Yamaguchi, T. Mechanical oscillation coupled with the Belousov-Zhabotinsky reaction in gel. *Langmuir* **19,** 5595-5600 (2003).
3. Szilagyi, A. *et al.* Rewritable microrelief formation on photoresponsive hydrogel layers. *Chem. Mater.* **19,** 2730-2732 (2007).
4. Satoh, T., Sumaru, K., Takagi, T., Kanamori, T. Fast-reversible light-driven hydrogels consisting of spirobenzopyran-functionalized poly(N-isopropylacrylamide). *Soft Matter* **7,** 8030-8034 (2011).
5. Yamamoto, T., Yoshida, R. Self-oscillation of polymer and photo-regulation by introducing photochromic site to induce LCST changes. *React. Funct. Polym.* **73,** 945-950 (2013).

6. Yashin, V. V., Balazs, A. C. Theoretical and computational modeling of self-oscillating polymer gels. *J. Chem. Phys.* **126,** 124707 (2007).
7. Hirotsu, S. Softening of bulk modulus and negative Poisson's ratio near the volume phase transition of polymer gels. *J. Chem. Phys.* **94,** 3949-3957 (1991).
8. Satoh, T., Sumaru, K., Takagi, T., Takai, K., Kanamori, T. Isomerization of spirobenzopyrans bearing electron-donating and electron-withdrawing groups in acidic aqueous solutions. *Phys. Chem. Chem. Phys.* **13,** 7322-7329 (2011).
9. Kuksenok, O., Balazs, A. C. Modeling the Photoinduced Reconfiguration and Directed Motion of Polymer Gels. *Adv. Funct. Mater.* **23,** 4601-4610 (2013).
10. Kuksenok, O., Yashin, V. V., Balazs, A. C. Three-dimensional model for chemoresponsive polymer gels undergoing the Belousov-Zhabotinsky reaction. *Phys. Rev. E* **78,** (2008).
11. Dayal, P., Kuksenok, O., Balazs, A. C. Using Light to Guide the Self-Sustained Motion of Active Gels. *Langmuir* **25,** 4298-4301 (2009).
12. Dayal, P., Kuksenok, O., Balazs, A. C. Designing autonomously motile gels that follow complex paths. *Soft Matter* **6,** 768-773 (2010).
13. Lu, X. *et al.* Photophobic and phototropic movement of a self-oscillating gel. *Chem. Commun. (Camb)* **49,** 7690-7692 (2013).
